# Event-related brain potentials reveal enhancing and compensatory mechanisms during dual neurocognitive and cycling tasks

**DOI:** 10.1186/s13102-023-00749-6

**Published:** 2023-10-17

**Authors:** Hsiao-Lung Chan, Yuan Ouyang, Cheng-Chou Lai, Ming-An Lin, Ya-Ju Chang, Szi-Wen Chen, Jiunn-Woei Liaw, Ling-Fu Meng

**Affiliations:** 1 Department of Electrical Engineering, Chang Gung University, Taoyuan, Taiwan; 2 Neuroscience Research Center, Chang Gung Memorial Hospital, Linkou, Taiwan; 3 Department of Neurology, Chang Gung Memorial Hospital, Linkou, Taiwan; 4 Faculty of Computer and Software Engineering, Huaiyin Institute of Technology, Huaian, Taiwan Jiang-Su; 5 School of Physical Therapy and Graduate Institute of Rehabilitation Science, College of Medicine, and Health Aging Research Center, Chang Gung University, Taoyuan, Taiwan; 6 Department of Electronic Engineering, Chang Gung University, Taoyuan, Taiwan; 7 Department of Mechanical Engineering, Chang Gung University, Taoyuan, Taiwan; 8 Center for Advanced Molecular Imaging and Translation, Chang Gung Memorial Hospital, Linkou, Taiwan; 9 Department of Occupational Therapy and Graduate Institute of Behavioral Science, College of Medicine, Chang Gung University, No.259, Wenhua 1st Rd., Guishan Dist, Taoyuan, 33302 Taiwan; 10 Division of Occupational Therapy, Department of Rehabilitation, Chiayi Chang Gung Memorial Hospital, Chiayi, Taiwan

**Keywords:** Dual tasks, Cycling, Electroencephalogram, Event-related brain potential, Color*–*word matching, Arithmetic calculation, Spatial working memory

## Abstract

**Background:**

Various neurocognitive tests have shown that cycling enhances cognitive performance compared to resting. Event-related potentials (ERPs) elicited by an oddball or flanker task have clarified the impact of dual-task cycling on perception and attention. In this study, we investigate the effect of cycling on cognitive recruitment during tasks that involve not only stimulus identification but also semantic processing and memory retention.

**Methods:**

We recruited 24 healthy young adults (12 males, 12 females; mean age = 22.71, SD = 1.97 years) to perform three neurocognitive tasks (namely color-word matching, arithmetic calculation, and spatial working memory) at rest and while cycling, employing a within-subject design with rest/cycling counterbalancing.

**Results:**

The reaction time on the spatial working memory task was faster while cycling than at rest at a level approaching statistical significance. The commission error percentage on the color–word matching task was significantly lower at rest than while cycling. Dual-task cycling while responding to neurocognitive tests elicited the following results: (a) a greater ERP P1 amplitude, delayed P3a latency, less negative N4, and less positivity in the late slow wave (LSW) during color-word matching; (b) a greater P1 amplitude during memory encoding and smaller posterior negativity during memory retention on the spatial working memory task; and (c) a smaller P3 amplitude, followed by a more negative N4 and less LSW positivity during arithmetic calculation.

**Conclusion:**

The encoding of color-word and spatial information while cycling may have resulted in compensatory visual processing and attention allocation to cope with the additional cycling task load. The dual-task cycling and cognitive performance reduced the demands of semantic processing for color-word matching and the cognitive load associated with temporarily suspending spatial information. While dual-tasking may have required enhanced semantic processing to initiate mental arithmetic, a compensatory decrement was noted during arithmetic calculation. These significant neurocognitive findings demonstrate the effect of cycling on semantic-demand and memory retention-demand tasks.

## Background

Engaging in cognitive tasks while being simultaneously involved in physical exercise might enhance cognitive functioning [1–3]. The mode of physical exercise differentially affects cognitive performance. For example, cycling enhanced cognitive performance during and after exercise, whereas treadmill running resulted in impaired cognitive performance during exercise but a slight improvement in cognitive performance after exercise [4].

The instant effects of exercise on cognitive performance were most demonstrated through the behavior responses to cognitive stimuli. A positive cognitive effect of exercise through the dual cognitive-cycling task paradigm was observed in individuals with Parkinson’s disease and healthy elderly people. Hazamy et al*.* reported that performing cognitive tasks while cycling more effectively facilitated reaction times to a visual attention task and recruited more verbal recalls during an executive function task in both participants with Parkinson’s disease and healthy older adults than performing these tasks when not cycling [5]. Chang et al*.* demonstrated that cycling improved cognitive performance (the ratio of accuracy to reaction time) on a calculation task in participants with Parkinson’s disease [6].

However, non-significant cognitive improvement or worse cognitive response was reported in several studies with healthy persons. For example, Bullock et al*.* reported the reaction time while seeing obliquely oriented faces during low-intensity exercise was not significantly different from that at rest, whereas only high-intensity cycling exercise produced faster detection response [7]. Similar findings were reported in the Eriksen flanker task while cycling [8]. What’s more, Lin et al*.* reported decreased response times to the visual attention task during vigorous cycling exercise [9]. These studies did not reveal higher accuracy of cognitive performance while cycling.

Searching for the evidence of cognitive processing directly from cerebral potentials provide a direct neural-related probe for determining the effect of exercise on cognitive functioning. Event-related potential (ERP) which is obtained by averaging electroencephalogram (EEG) readings over several trials with the same type of stimulus is widely used to investigate the brain response resulting from a cognitive stimulus event. In contrast to fMRI and other neuroimaging studies, EEG/ERP provides exceptional temporal resolution and it can be conducted promptly during cycling and demonstrates the instant effect on neurophysiological responses after cycling.

P1 and P3 are positive ERP components that occur approximately 100 and 300 ms after the stimulus event. They are generally associated with cognitive processing in response to a visual stimulus [10] and stimulus identification [11]. Modulation of the ERP components while simultaneous cognitive tasking and cycling was demonstrated in the literature, as summarized in Table 1. A study on ERP during an auditory oddball task revealed comparable P3 components when participants performed the task while cycling or sitting [12]. Bullock et al*.* further found that a visual oddball task elicited earlier, larger P1 and earlier P3 components when participants were cycling than when they were at rest [7]. However, Yagi et al*.* found that auditory and visual oddball tasks produced earlier decreased P3 components during cycling than at rest [13]. Other studies have found that the Eriksen flanker task produced higher N2 and P3 amplitudes [8, 14] but later N2 and P3 latencies [14] while cycling than at rest. Lin et al*.* found that the P3 amplitude for target letter identification seems higher while performing vigorous cycling the task [9]. In summary, dual-task cycling created earlier P3 latency on most oddball tasks but later P3 latency on the flanker task; larger P3 and N2 amplitude in most studies except smaller P3 amplitude on the auditory and visual oddball tasks. The possible mechanisms can be attributed to the following. The increased N2 and P3 amplitudes on the flanker task while cycling were regarded as the upregulation of cognitive control [8] and the need of attentional resources allocation toward body movements [14] inherent in cycling exercise, whereas the decreased P3 amplitude on the visual oddball task while cycling suggested the diminished attentional resource allocation [13]. On the other hand, the later latency on the flanker task while cycling compared to the earlier latency on the visual oddball task while cycling was attributed to a greater demand for cognitive control on the flanker task [7].

**Table 1:** Dual cognitive-cycling tasking event-related potential literature review

Reference	Cognitive task	Latency	Amplitude
Scanlon et al*.* (2017) [12]	Auditory oddball	Non-significant	Larger P3
Bullock et al*.* (2015) [7]	Visual oddball	Earlier P3	Larger P1
Yagi et al*.* (1999) [13]	Auditory & visual oddball	Earlier P3	Smaller P3
Pontifex & Hillman (2007) [14]	Eriksen flanker	Later N2 Later P3	Larger N2 Larger P3
Olson et al*.* (2016) [8]	Eriksen flanker	Non-significant	Larger N2 Larger P3
Lin et al*.* (2021) [9]	Target letter identification	Non-significant	Larger P3

Peak latency and peak amplitude while cycling are compared with those at rest

Most of these researchers demonstrate that stimulus identification is mediated, depending on the task type, when participants are executing cycling activity simultaneously. Nevertheless, the aforementioned oddball tasks, the flanker task, and target letter identification task are more associated with the basic attention and perceptual capacity at the non-conceptual and non-semantic level [13, 15]. The cycling effect on the task performance at the conceptual and semantic level such as judgement, calculation, and working memory are only investigated by behavior performance [5, 6] but have not been studied on the basis of ERP measures.

The color–word matching task has been used for the judgment whether the meaning of a word is matched to its color. Several researchers have demonstrated the presence of several ERP components such as P3, N4 and late negative wave by this task [16–18], which correspond to response inhibition [17] and semantic selection [16]. Mental arithmetic task has been used to investigate the problem-solving strategy based on the induced late positive slow wave, which starts at about 400 ms after stimuli, in the ERP. The slow wave is modulated by the problem size and arithmetic strategy [19, 20]. On the other hand, spatial working memory task is used to examine a delayed match-to-sample task in which spatial information is encoded and retained in a working memory for a period for comparison with a subsequent stimulus. The increased negative slow wave in ERP during memory retention is associated with increasing spatial memory load [21].

The aforementioned dual-task effects involved with judgement, working memory and semantic processing are assessed by cognitive performance [5, 6], whereas the dual-task effects on the cerebral operations for these tasks remain unexamined. With a strong motivation to connect instant effects and brain mechanisms via detailed EEG/ERP experiments, our current study extends from the mentioned cognitive performance to neurophysiological responses on the calculation and two executive function experiments—working memory and color–word matching. At the behavioral and neurocognitive level, this study examines whether simultaneous cycling produces neurophysiological effects similar to those seen in neurobehavioral performance and whether neural resources are reallocated to handle dual-task workload in these domains. We recruited healthy young adults to perform two semantic-demand tasks (a color–word matching task and an arithmetic calculation task) and one memory retention-demand task (a spatial working memory task) while cycling on a stationary bicycle and while at rest. Dual-task effects on neurocognitive functions were therefore investigated by comparing the behavioral responses and ERPs between dual cognitive–cycling task and single cognitive task, where these comparisons have also been used to understand the impact of physiological recruitment by aging and disease [22–24]. Compared to single cognitive task, dual cognitive–cycling task create an additional motor work and may produce enhancing or complementary mechanisms at the conceptual and semantic levels as well as the mechanisms at basic attention and perceptual levels [7, 8, 12–14]. Therefore, we hypothesized that cycling during color–word matching, spatial working memory, and arithmetic calculation tasks would result in changes to reaction time, response accuracy and ERP measures (P1, P3, semantic- and memory retention-related components) in comparison to resting.

## Materials and methods

### Participants

We determined the required sample size through a priori statistical power analysis by using G*Power 3 software [25]. In this calculation, we assumed a two-level task type with single and dual tasks, pairwise *t* tests, a need for 80% statistical power, α = 0.05, and a Cohen’s effect size of 0.60. The sample size was calculated as 24 participants.

Effect sizes of 0.60 or greater have been reported in previous studies. Both reported large Cohen’s d values despite small sample sizes [26, 27]. Zeng et al*.* [27] recruited twelve healthy college students who completed two distinct 20-min exercise sessions respectively on a virtual reality (VR) based exercise bike and a traditional stationary exercise bike. Dependent t-tests revealed that participants reported notably elevated perceived exertion ratings (*p* < 0.05, Cohen's d = 0.68) during the traditional exercise biking session compared to the VR-based exercise biking session. However, participants exhibited significantly increased self-efficacy (*p* < 0.05, Cohen's d =  − 0.83) and enjoyment (*p* < 0.05, Cohen's d =  − 0.89) during the VR-based exercise biking session in contrast with traditional stationary biking. All three Cohen's d values exceeded 0.6, despite the study's small sample size of only 12 participants.

Jensen and Kenny [26] found the significant effects for 11 children with attention-deficit/hyperactivity disorder participating the yoga group on 5 subscales of the Conners’ Teacher Rating Scale: Oppositional (*p* = 0.003, Cohen’s d = 0.77), Global Index Emotional Lability (*p* = 0.001, Cohen’s d = 0.79), Global Index Total (p = 0.001, Cohen’s d = 0.73), and Global Index Restless/ Impulsive (p = 0.008, Cohen’s d = 0.73). Despite the participation of only 11 individuals, all four Cohen's d values surpassed 0.7.

We therefore recruited 24 healthy, right-handed young adults (12 males, 12 females; mean age = 22.71, *SD* = 1.97 years; mean height = 165.20, *SD* = 6.50 cm; mean weight = 57.30, *SD* = 13.19 kg).

### Procedure

Each participant visited lab once for conducting neurocognitive tasks in rest and cycling conditions. Each participant sat on a cycle ergometer (XTERRA SB3.5, Dyaco International, Taiwan), which was located approximately 1.5 m from a 32-inch liquid crystal display monitor. The monitor was placed such that the participant could visualize the virtual reality-guided cognitive test images in a straight angle.

Each participant was asked to sit in an upright position and perform each virtual reality-guided neurocognitive task under two conditions: at rest and while cycling. Each participant was asked to sit in an upright position and perform each virtual reality-guided neurocognitive task under two conditions: at rest and while cycling. The order of rest or cycling was counterbalanced across participants; half of the participants performed the neurocognitive tasks at rest first, and the other half performed the tasks while cycling first. Each participant rested for 3 min after each block.

### Virtual reality–guided neurocognitive tasks during rest and cycling

The three neurocognitive tasks included a color–word matching task, an arithmetic calculation task, and a spatial working memory task, with each task administered in a virtual reality environment developed using Unity3D (Unity Technologies, San Francisco, CA, USA). The order of these three paradigms was counterbalanced across the participants. The participants were instructed to perform these cognitive tasks while cycling (dual tasks) and at rest (single task). To reduce the possibility of muscle fatigue during cycling, light-intensity cycling with a target power of 30 W was adopted.

A pedaling sensing module based on a microcontroller (MSP430F5438, Dallas, TX, USA), 6-axial inertial chip (LSM330DLC, STMicroelectronics, Geneva, Switzerland), and a Bluetooth module (BTC-1022, Atech OEM Inc., Taipei, Taiwan) was used to measure and wirelessly transmit the pedaling speed to the Unity program. A small thin vertical bar was displayed at the center of the road to indicate the pedaling speed. The participant was instructed to execute the cycling task by controlling the cursor in the middle of the bar, which was equivalent to a pedaling speed of 40 revolutions per minute (rpm) while performing a cognitive task. During rest, the participant was instructed to not pedal the bicycle but performed a cognitive task; in this situation, the road still moved at a constant speed corresponding to a pedaling speed of 40 rpm.

### Color–word matching task

**Fig. 1:**
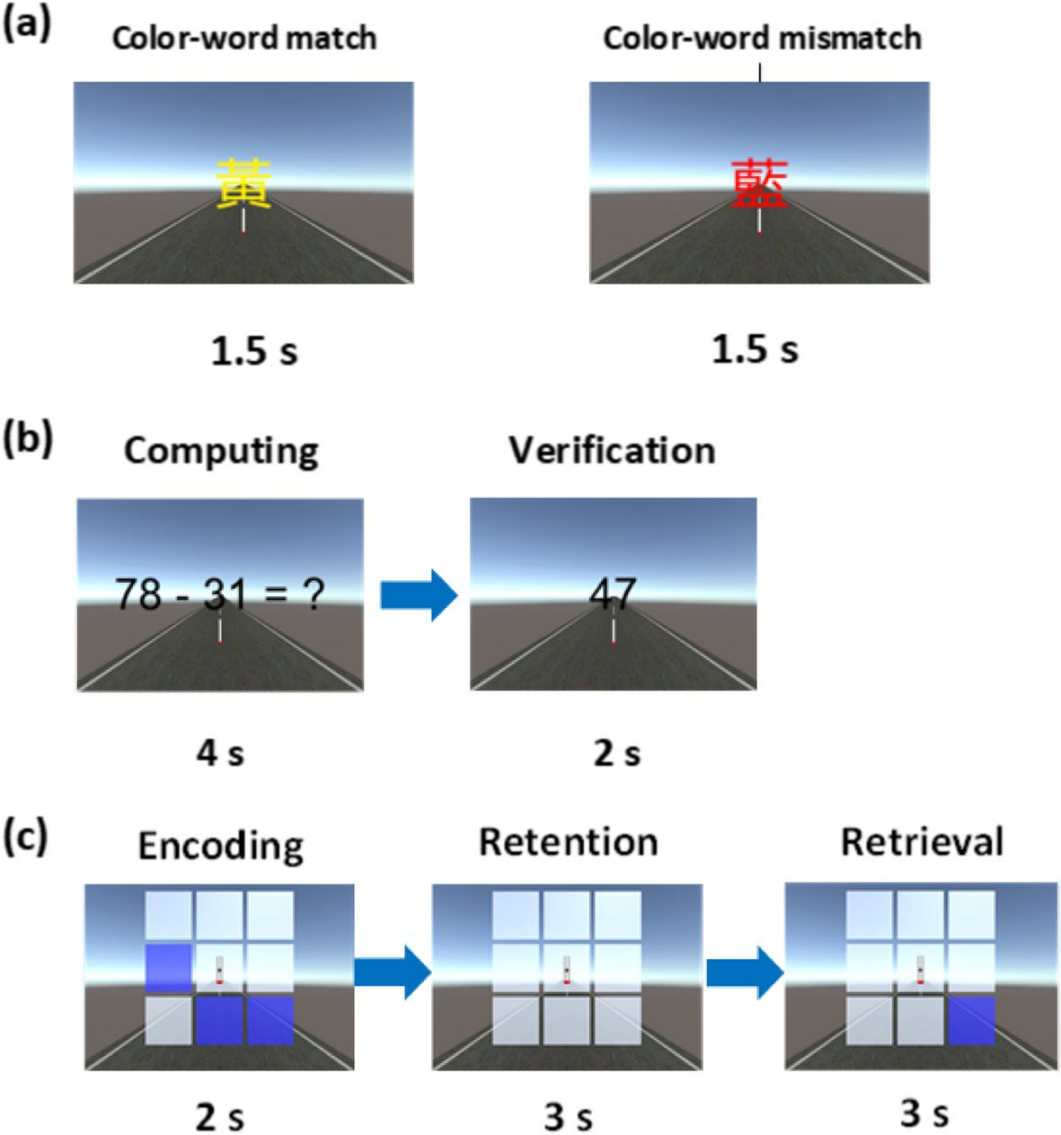
(**a**) The Color–Word Matching Task: The Chinese color word matched the meaning of the word (黃 on the left) in one trial but did not match in another trial (藍 on the right). **b** The Arithmetic Calculation Task. **c** The Spatial Working Memory Task: The depiction illustrates an example of a matched presentation in encoding and retrieval periods

Figure 1a illustrates the color–word matching task. In this task, three Chinese words meaning red (紅), blue (藍), and yellow (黃) were randomly displayed on the screen for 1.5 s. While the task included 120 trials, 88% of these trials were color–word matches in which the color of the letters matched the color word shown (i.e., the word “green” was presented in green font). The remaining 12% trials were color–word mismatched. The participants had to press a button when they saw a mismatched presentation, requiring them to be extremely attentive. The inter-trial interval was set to 2 s. The time to complete the total 120 trials was 7 min for each participant.

### Arithmetic calculation task

Figure 1b illustrates the arithmetic calculation task. This task also included 120 trials, with each task presenting a two-digit subtraction problem for 4 s. In 96 trials, there was no need to borrow from the “tens” column, and in 24 subtraction trials, there was a need to borrow. Borrowing problems were involved to increase the calculation challenge. An answer was subsequently presented for 2 s, with the correct answer provided for 88% of the 120 trials and an incorrect answer provided for the rest. The participants were instructed to press a button when an incorrect answer was presented. The inter-trial interval was set to 2 s. The time to complete the total 120 trials was 16 min for each participant.

### Spatial working memory task

Figure 1c illustrates the spatial working memory task containing encoding, retention, and retrieval periods. In each trial, a 3 × 3 array of squares was colored in white before the encoding period. Three randomly chosen squares from among the outer eight squares were colored in blue, and the presentation lasted for 2 s during the encoding period. In the subsequent retention period, all blue-colored squares were restored to white to force the participant to memorize the previous blue-colored squares for 3 s. After entering the retrieval period, one of the eight outer squares was colored in blue, and the participant had to judge whether this blue-colored square matches one of the three blue-colored squares that had appeared in the encoding period. Of the 120 trials, 88% of the trials displayed blue-colored squares in the retrieval period that matched those displayed in the encoding period, whereas the remaining 12% trials presented blue-colored squares that did not match. The participants were instructed to press a button for mismatched trials. The inter-trial interval was set to 2 s. The time to complete the total 120 trials was 20 min for each participant.

### Data collection

We used a passive electrode cap which contains 32 scalp electrodes according to the international 10/20 system: Fp1, Fp2, F7, F3, Fz, F4, F8, FC5, FC1, FC2, FC6, T7, C3, Cz, C4, T8, CP5, CP1, CP2, CP6, P7, P3, Pz, P4, P8, O1, O2, VEOG, LEOG, and REOG (LiveCap, Brain Vision, Garner, NC). A wireless 32-channel, 24-bit EEG amplifier (LiveAmp 32, Brain Products, Gilching, Germany) was used to obtain EEG recordings with an impedance check of < 10 KΩ with respect to a reference electrode FCz at a sampling rate of 500 Hz. The recorded EEG channels were re-referenced to the average mastoids. The referenced EEG signals were detrended using a high-pass filter with a cutoff frequency of 0.5 Hz and denoised using a low-pass filter with a cutoff frequency of 30 Hz. EEG data were gathered from each participant during single- and dual-task activities. The time for performing color–word matching, arithmetic calculation, and spatial working memory tasks while cycling or at rest were about 7, 16, and 20 min. The impedance was therefore checked before each experimental task.

### Behavioral analysis

In this study, participants pressed the button only in the situation while seeing incorrect trials. Hence, the accuracy score and reaction time while responding to the incorrect trials were treated as the behavioral capacity. On the color–word matching task, the participants’ reaction times were the duration from the word presentation to pressing of the button for mismatched trials. On the arithmetic calculation task, the reaction time was the duration from answer presentation to pressing of the button for mismatched trials. On the spatial working memory task, the reaction time was the duration from the presentation of the blue-colored square in the retrieval period to pressing of the button for mismatched trials. In our experiment, a mere 12% of the total trials involved mismatched presentations, during which participants were instructed to press a button. Given that a limited number of mismatched trials were incorporated into the computation of reaction time and this computation demonstrated a consistent adherence to normality, as confirmed by the Shapiro–Wilk test, we refrained from excluding outliers during the reaction time calculation. The omission error was the percentage of no-button-press trials relative to all mismatch trials. The commission error was the percentage of incorrect-button-press trials relative to all match trials.

### ERP analysis

Independent component analysis was performed to decompose multi-channel EEGs into a equivalent number of source components using EEGLAB [28]. The identified source components that were related to ocular or high-frequency muscular or low-frequency movement artifacts were excluded for reconstruction. The reconstructed EEGs were segmented according to the selected events in various neurocognitive tasks. The trials with amplitudes exceeding ± 80 μV were further rejected to reduce the effect of other possible artifacts on the ERP computation, which has been used to exclude EEGs with movement artifacts [29–31]. Therefore, the number of the matched trials with maximal amplitude less than 80 μV used to compute ERP at rest and while cycling was 86.3 ± 7.8 and 86.9 ± 6.8, respectively (*p* = 0.753) on the color–word matching task, 84.1 ± 6.1 and 87.2 ± 5.4, respectively (*p* = 0.027) on the arithmetic calculation task, and 90.5 ± 7.0 and 92.2 ± 6.0, respectively (*p* = 0.261) on the spatial working memory task.

In general, the 200-ms interval before each prime stimulus was used as a baseline. ERPs were obtained from the average over the baseline-subtracted potentials from the matching trials that were correct. During the color–word matching task, the 200-ms interval before word presentation was set as the baseline. The 1200-ms post-stimulus interval was used for ERP calculation. Four ERP components were obtained after baseline subtraction. P1 was the most positive component from 170 to 230 ms after word presentation, and P3a was from 200 to 320 ms. N4 was the most negative component from 300 to 500 ms. Late slow wave (LSW) was quantified by the mean potential from 800 to 1000 ms. These latency windows were set based on the neurocognitive-related components observed in the grand-averaged ERP.

During the arithmetic calculation task, the 200-ms interval before question presentation was set as the baseline. The 2-s post-stimulus interval was used for ERP calculation. P3 was the most positive component from 200 to 400 ms after the onset of computing, and N4 the most negative component from 400 to 600 ms. LSW was quantified using the mean potential from 400 to 700 ms.

During the spatial working memory task, the 200-ms interval before the onset of the encoding period (occurrence of blue-colored squares) was set as the baseline. The 5-s post-stimulus interval composed of 2-s encoding period and 3-s retention period was used for ERP calculation. P1 was obtained as the most positive component from 150 to 200 ms after the onset of encoding. Since both a 2-s retention period and a longer period (5 s) produced significant retention effects on the ERP in a spatial working memory task [32], we therefore divided the 3-s retention period into early (the 1^st^ second), middle (the 2^nd^ second), and late (the 3^rd^ second) stages to study the effect of the sustained retention on the ERP in our spatial working memory task.

Six cerebral areas were defined over cerebral electrodes: left centroposterior (F3, F7, FC5, C3), centrofrontal (Fz, FC1, FC2, Cz), right centroposterior (F4, F8, FC6, C4), left centroposterior (CP1, CP5, P3, P7), posterior (Pz, O1, O2), and right centroposterior (CP2, CP6, P4, P8). Based on the findings of previous researchers including significant P3 and late negative wave in the centrofrontal area on the color–word matching task [16, 18], significant negative slow wave in the posterior and centroposterior areas during the retention period of spatial working memory task [32], and significant positive slow wave from posterior to central areas while solving mathematical problem [20]. The ERP of each area was computed by averaging the ERPs measured from the defined electrodes. The aforementioned ERP components were obtained from the regional ERPs. The amplitudes and latencies of these components were used to study the effect of cycling on neurocognitive responses.

### Statistical analysis

Reaction time and accuracy obtained during various neurocognitive tasks were used to study the effect of cycling versus rest on behavior responses. The data were determined to be normal by using the Shapiro–Wilk test. The paired *t* test was used to compare differences between reaction time performances at rest and while cycling. The effect size (ES) is expressed by Cohen’s d. The size of the difference related to the variance of the variable is measured by *t* value with degrees of freedom of 23. The statistical significance is shown by *p* value. The Wilcoxon signed-rank test was used to compare the omission and commission error differences between performances at rest and while cycling because these data differed significantly from the normal. Their effect size calculations involved dividing the Z-score by the square root of the case number (*N* = 24).

For P1, P3, and N4 components, peak amplitude was defined as the maximal amplitude, and peak latency was defined as the duration from stimulus to the peak time. The values of LSW or PN were averaged as its representative amplitude. Peak amplitudes, peak latencies, average LSW, and average PN obtained during various neurocognitive tasks were used to study the effect of cycling versus rest on neurocognitive responses. The differences in these parameters between rest and cycling were analyzed using a paired t test with *p* < 0.05 considered as statistically significant using MATLAB R2015b (The MathWorks, Natick, MA). The size of the difference related to the variance of the variable is measured by *t* value with degrees of freedom of 23. The Cohen’s effect size for the paired t test was calculated by dividing the mean difference by the standard deviation of the difference. Due to the high number of multiple comparisons, we used the sequential Benjamini–Hochberg correction after conducting multiple t-tests for the purpose of preventing the type I error (alpha inflation). In addition, repeated measure analysis of variance (ANOVA) was used to analyze the mean ERPs over retention stages (early, middle, and late) on the spatial working memory task, and multiple Bonferroni tests were applied for post hoc pairwise comparisons using IBM SPSS Statistic 22 (International Business Machines Corporation, Armonk, NY).

## Results

### Behavioral results on neurocognitive tasks while at rest and while cycling

Table 2 presents the statistical results of participants’ reaction times, omission errors, and commission errors which were calculated based on the mismatched trials at rest and while cycling. The reaction time on the spatial working memory task tended to be faster while cycling than at rest (*t*(degrees of freedom) = 1.948, *p* = 0.0604). The commission error percentage on the color–word matching task was significantly lower at rest than while cycling (*p* = 0.042). No other behavioral results on neurocognitive tasks were significant.

**Table 2:** Participants’ neurocognitive behavioral performances while cycling and at rest

Variable	Color*–*word matching	Arithmetic calculation	Spatial working memory
Reaction time, ms			
Rest	468.9 ± 65.7	534.4 ± 107.5	523.0 ± 143.0
Cycling	459.5 ± 56.1	509.8 ± 90.2	480.6 ± 81.6
Effect size	0.203	0.192	0.398
*t*	0.994	0.942	1.948
*p* value	0.330	0.356	0.064
Omission error, %			
Rest	5.514 ± 5.556	5.213 ± 6.825	2.795 ± 6.020
Cycling	2.925 ± 4.987	5.765 ± 8.491	1.854 ± 5.404
Effect size	2.925 ± 4.987	0.054	0.085
*p* value	0.114	0.794	0.678
Commission error, %			
Rest	0.042 ± 0.206	1.292 ± 1.107	0.708 ± 1.781
Cycling	0.309 ± 0.669	1.313 ± 1.278	0.832 ± 1.334
Effect size	0.415	0.053	0.050
*p* value	***0.042***	0.794	0.807

All data are presented as mean ± standard deviation

### ERP results on the color–word matching task

**Fig. 2:**
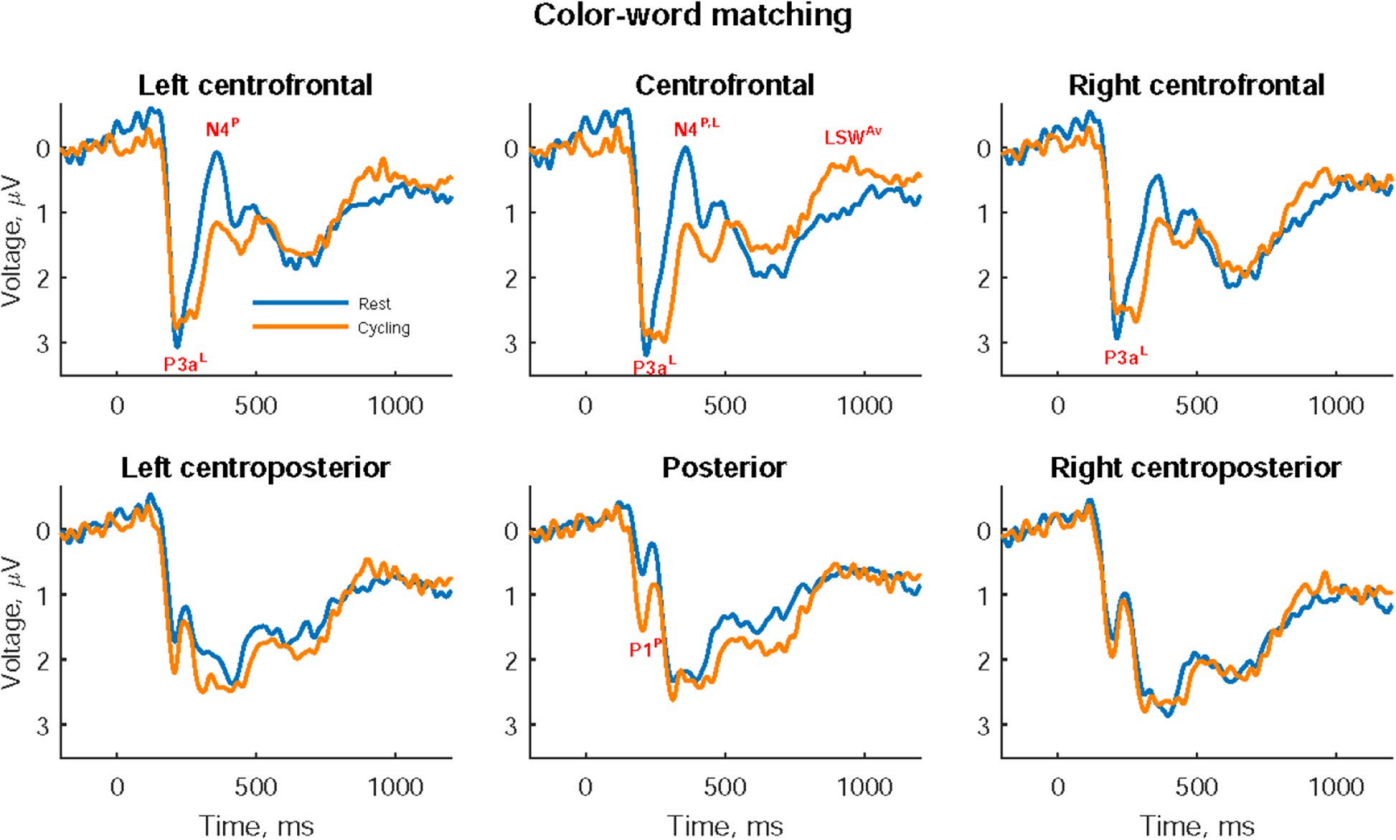
Event-related potentials on the color–word matching task. Significant P1 peak amplitude (P1^P^), significant P3a peak latency (P3a^L^), significant N4 peak amplitude (N4^P^), significant N4 peak latency (N4^L^), and significant average late slow wave (LSW^Av^) between rest and cycling are marked in their corresponding cerebral area

As shown in Fig. 2, different ERP patterns were presented with a distinct P1 component in the posterior area and distinct N4, LSW in the centrofrontal area. Table 3 presents the statistical results of participants’ ERPs at rest and while cycling. Since the centrofrontal area displayed an obvious LSW which distributed over a longer duration than P1, P3a, and N4 components, the amplitudes from 800 to 1000 ms were averaged to convey its slow-varying characteristic. Although posterior P1 and centrofrontal P3a seem to share latency in Fig. 2, posterior P1 peak had a latency of 203.2 ± 20.9 ms while cycling, occurring earlier than centrofrontal P3a peak with a latency of 263.6 ± 37.0 ms while cycling. In summary, the P1 peak amplitude in the posterior area was significantly greater while cycling than at rest (*t* = -2.244, *p* = 0.0348). ERPs in the centrofrontal area showed a significantly delayed P3a peak latency (*t* = -3.315, *p* = 0.0030), followed by a significantly smaller, earlier N4 peak amplitude (*t* = 2.278, *p* = 0.0324) and a smaller positive LSW while cycling than at rest (*t* = 2.500, *p* = 0.0200).

**Table 3:** Participants’ event-related potentials (ERPs) on the color–word matching task

**ERP Component Area**	**Amplitude, μV**					**Latency, ms**				
	**Rest**	**Cycling**	**ES**	***t***	***p***	**Rest**	**Cycling**	**ES**	***t***	***p***
**P1**										
Posterior	1.72 ± 2.20	2.57 ± 2.42	0.458	-2.244	***0.0348***	202.7 ± 20.3	203.2 ± 20.9	0.020	-0.099	0.9216
**P3a**										
Left centrofrontal	4.18 ± 2.80	4.23 ± 2.96	0.021	-0.102	0.9194	241.2 ± 35.8	258.0 ± 36.8	0.467	-2.290	***0.0315***
Centrofrontal	4.38 ± 3.50	4.53 ± 2.30	0.057	-0.278	0.7832	239.0 ± 35.1	263.6 ± 37.0	0.677	-3.315	***0.0030***
Right centrofrontal	4.03 ± 2.70	4.07 ± 2.49	0.023	-0.112	0.9115	243.9 ± 42.3	268.8 ± 38.4	0.528	-2.587	***0.0165***
**N4**										
Left centrofrontal	2.30 ± 2.22	3.09 ± 1.73	0.396	-1.942	0.0645	418.8 ± 72.5	352.2 ± 69.6	0.880	4.312	***0.0003***
Centrofrontal	2.46 ± 2.47	3.41 ± 1.93	0.457	-2.238	***0.0352***	405.8 ± 77.6	361.9 ± 93.2	0.465	2.278	***0.0324***
**LSW**										
Centrofrontal	1.03 ± 1.31	0.42 ± 1.21	0.510	2.500	***0.0200***					

All data are presented as mean ± standard deviation. The paired *t* test was used to compare differences between ERP peaks’ amplitudes and latencies at rest and while cycling and differences between the mean amplitude of late slow wave (LSW) at rest and while cycling. The bold *p* value indicates significant variables after conducting Hochberg’s correction on each component

### ERP results on the arithmetic calculation task

**Fig. 3:**
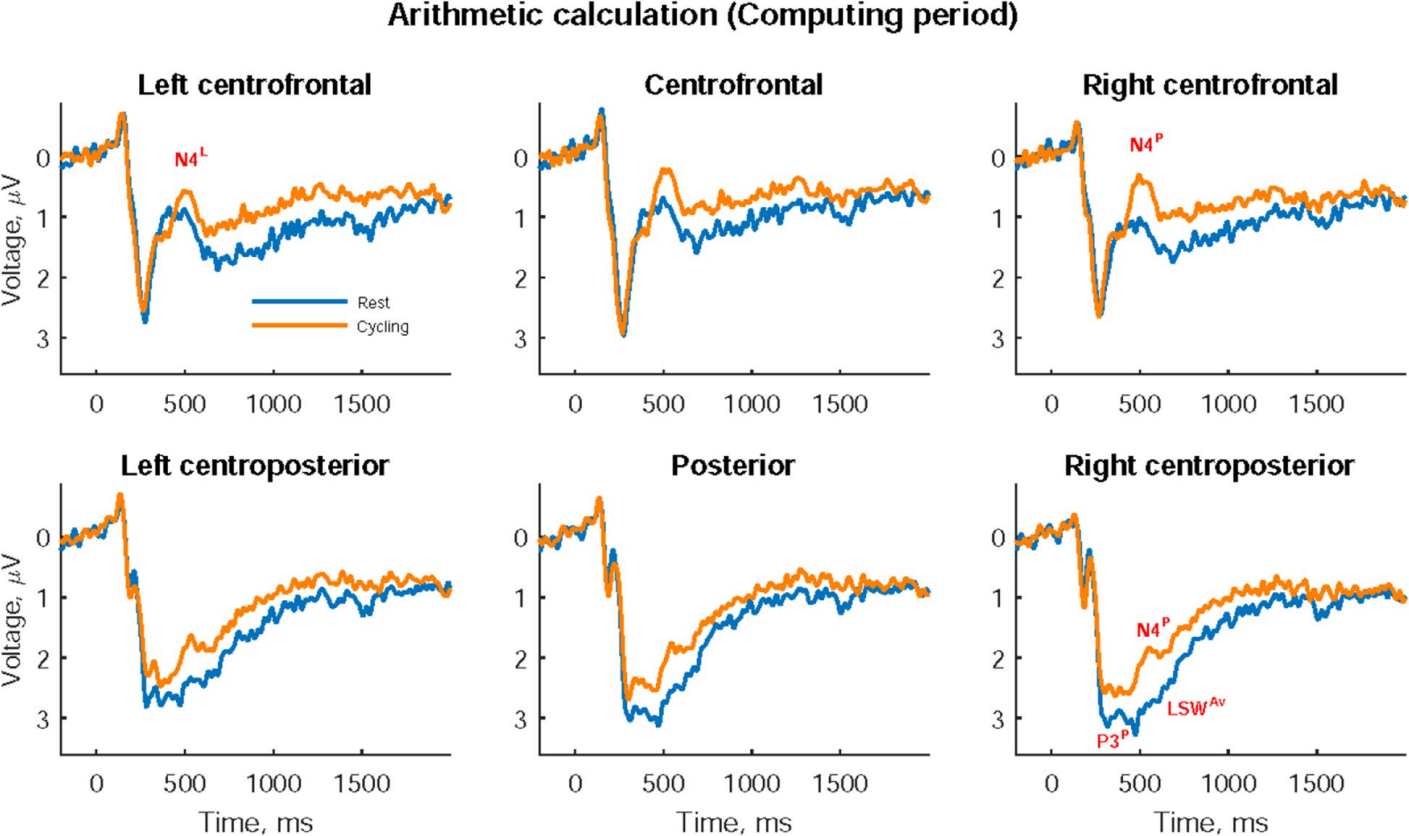
Event-related potentials during the computing period on the arithmetic calculation task. Significant P3 peak amplitude (P3^P^), significant N4 peak amplitude (N4^P^), significant N4 peak latency (N4^L^), and significant average late slow wave (LSW^Av^) between rest and cycling are marked in their corresponding cerebral area

Figure 3 shows participants’ ERPs during the computing period (after the presentation of the subtraction problem), with the statistical results of these ERPs at rest and while cycling presented in Table 4. The P3 peak amplitude in the right centroposterior area was significantly smaller while cycling than at rest (*t* = 2.157, *p* = 0.0417), but N4 peaks were significantly greater in the right hemisphere (*t* = 2.537, *p* = 0.0184) and occurred earlier in the left centrofrontal area (*t* = 2.220, *p* = 0.0365) while cycling than at rest. The subsequent LSW in the right centroposterior area was less positive while cycling compared with at rest (*t* = 2.415, *p* = 0.0241).

**Table 4:** Participants’ event-related potentials (ERPs) on the arithmetic calculation task

**Period/Component Area**	Amplitude, μV					Latency, ms				
	**Rest**	**Cycling**	***ES***	***t***	***p***	**Rest**	**Cycling**	***ES***	***t***	***p***
**Computing/P3**										
Right centroposterior	3.80 ± 2.58	3.21 ± 2.81	0.440	2.157	***0.0417***	287.6 ± 32.5	286.9 ± 37.9	0.014	0.069	0.9459
**Computing/N4**										
Left centrofrontal	2.45 ± 2.30	2.01 ± 2.36	0.320	1.569	0.1302	521.2 ± 80.2	480.7 ± 75.9	0.453	2.220	***0.0365***
Right centrofrontal	2.50 ± 2.24	1.86 ± 2.08	0.468	2.293	***0.0313***	490.5 ± 73.3	464.9 ± 74.7	0.304	1.491	0.1496
Right centroposterior	4.05 ± 2.78	3.21 ± 2.52	0.518	2.537	***0.0184***	496.8 ± 59.4	476.0 ± 70.2	0.273	1.339	0.1935
**Computing/LSW**										
Right centroposterior	2.78 ± 2.22	2.09 ± 1.89	0.493	2.415	***0.0241***					

All data are presented as mean ± standard deviation. The paired *t* test was used to compare differences between ERPs’ peak amplitudes and latencies at rest and while cycling and differences between the mean amplitude of late slow wave (LSW) at rest and while cycling. The bold *p* value indicates significant variables after conducting Hochberg’s correction on each component

### ERP results on the spatial working memory task

**Fig. 4:**
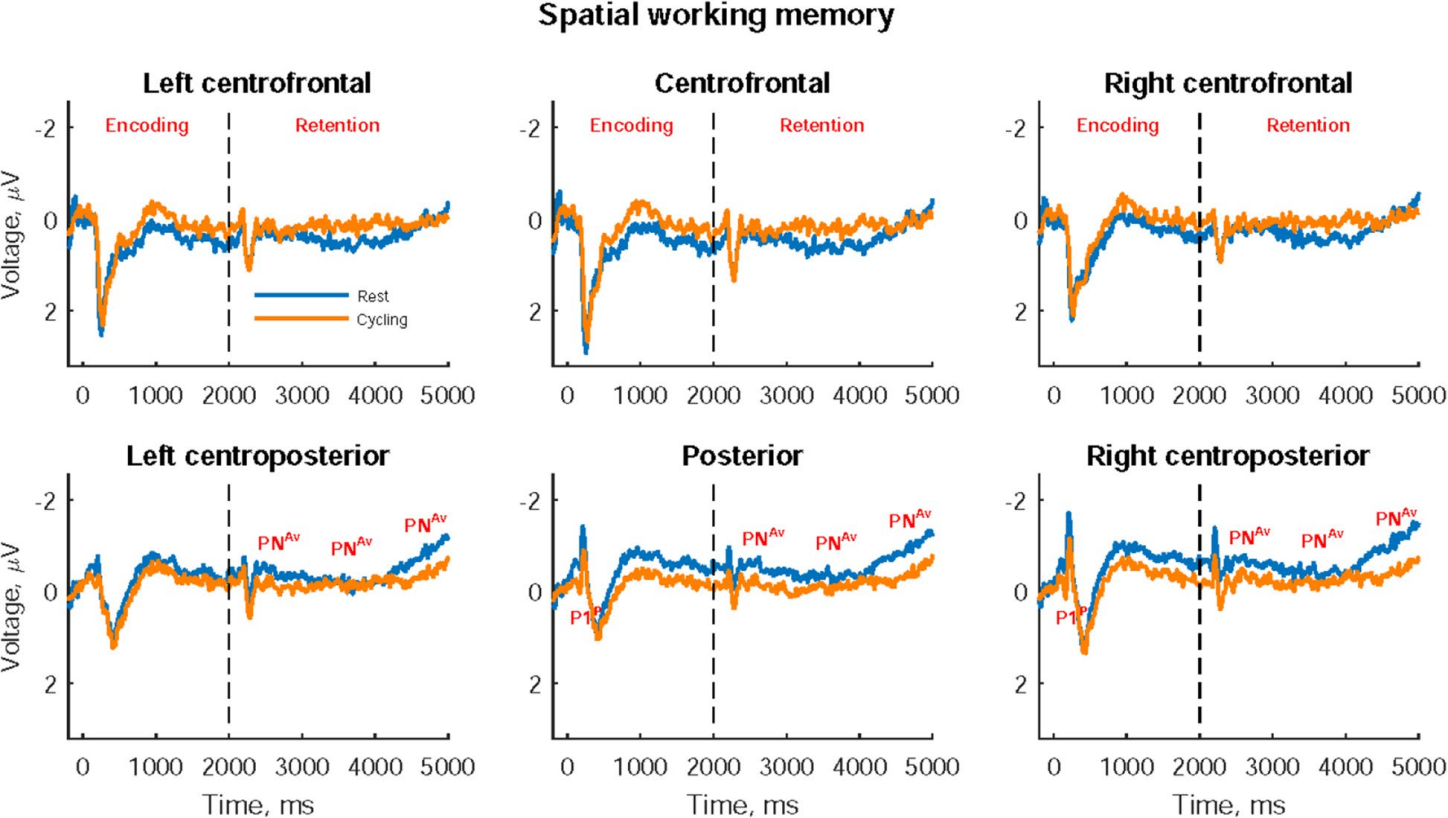
Event-related potentials on the spatial working memory task. Significant P1 peak amplitude (P1^P^) and significant average posterior negativity (PN^Av^) between rest and cycling are marked in their corresponding cerebral area

Figure 4 presents participants’ ERPs on the spatial working memory task, with significant P1 during the encoding period and negative-going ERP from the middle of the retention period. Table 5 presents the statistical results of these ERPs at rest and while cycling. We found significantly greater P1 peak amplitude in the right centroposterior areas while cycling than at rest (*t* = -2.172, *p* = 0.0404). However, significantly larger posterior negativity (PN) was observed during early-, mid-, and late-retention stages at rest than while cycling (*t* = -2.092, *p* = 0.0477; *t* = -2.368, *p* = 0.0267; *t* = -2.376, *p* = 0.0262); in particular, significant augmentation of PN was observed during the late-retention stage at rest.

**Table 5:** Participants’ event-related potentials (ERPs) on the spatial working memory task

**Period/Component Area**	Amplitude, μV					Latency, ms				
	**Rest**	**Cycling**	**ES**	***t***	***p***	**Rest**	**Cycling**	**ES**	***t***	***p***
**Encoding/P1**										
Posterior	0.04 ± 0.88	0.48 ± 0.68	0.423	-2.073	*0.0496*	168.0 ± 23.7	166.3 ± 21.8	0.049	0.242	0.8111
Right centroposterior	0.06 ± 0.89	0.50 ± 0.71	0.443	-2.172	***0.0404***	167.5 ± 19.6	162.0 ± 20.0	0.200	0.978	0.3381
**Early-retention/PN**										
Posterior	-0.46 ± 0.71	-0.09 ± 0.64	0.427	-2.092	***0.0477***					
Right centroposterior	-0.60 ± 0.87	-0.21 ± 0.78	0.422	-2.072	*0.0497*					
**Mid-retention/PN**										
Posterior	-0.36 ± 0.60	-0.03 ± 0.66	0.427	-2.094	*0.0475*					
Right centroposterior	-0.47 ± 0.66	-0.12 ± 0.73	0.483	-2.368	***0.0267***					
**Late-retention/PN**										
Posterior	-0.68 ± 0.71^a,b^	-0.27 ± 0.73	0.445	-2.179	*0.0398*					
Right centroposterior	-0.83 ± 0.78^a,b^	-0.36 ± 0.86	0.485	-2.376	***0.0262***					

All data are presented as mean ± standard deviation. The paired *t* test was used to compare differences between P1’s peak amplitudes and latencies at rest and while cycling and differences between posterior negativity (PN) and the mean amplitude of late slow wave at rest and while cycling. A repeated measures ANOVA was used to analyze PN over all retention stages, with post hoc pairwise comparisons performed using multiple Bonferroni tests. ^a^ and ^b^ separately indicate significantly greater PN during the late-retention stage than during the early- and mid-retention stages (*p* < 0.05). The **bold *p* value** indicates significant variables after conducting Hochberg’s correction on each component

## Discussion

### Behavioral cognitive responses

This study investigated the dual-task effect on cognitive responses including reaction time, omission error and commission error by using the color–word matching task, arithmetic calculation task, and spatial working memory task.

### Reaction time on the spatial working memory task

Our result revealed that only time taken to respond to the mismatched blue square on the spatial working memory task tended to be faster while cycling than at rest (*t* = 1.948, *p* = 0.0604). Some studies have demonstrated that dual-task cycling reduced reaction time compared with a single cognitive task [5, 7, 8, 13], but no effect of dual task on reaction time was also reported on a study [14]. Lambourne et al*.* concluded that exercise-induced arousal enhanced performance in tasks involving rapid decisions [4]. The evaluation of cognitive behavior response is limited in our study because the reaction time and accuracy were calculated based on a small number of unmatched trials (12% of all trials). Nevertheless, the cognitive facilitation by cycling-induced arousal seems take effect on the retrieval of spatial information, accompanying with efficient utilization of memory storage during retention period while cycling relative to rest, discussed in a later paragraph.

### Response accuracy on the color-word matching task

Our study on the color–word matching task showed more commission errors while cycling compared with the resting condition. The worse accuracy response is similar to the results of the flanker [8] and calculation tasks [33]. Olson et al*.* used transient hypofrontality theory [8], whereas Herold et al*.* adopted the limited resource hypothesis [33] to explain worse accuracy while exercising. The transient hypofrontality theory proposed by Olson et al*.* explained the decrease in response accuracy arising from exercise. The initiation and maintenance of exercise could constrain metabolic resources in brain regions that do not primarily respond to exercise, thus leading to reduced activity, including hypofrontality [8]. Furthermore, the limited resource hypothesis adopted by Herold et al*.* elaborated that cognitive resources become constrained during resistance exercise. In situation when both tasks' demanding is not satisfied simultaneously, the immediate demands of acute exercise hold priority [33]. These explanations can be used to deliberate our finding. In addition, the commission error was linked to response inhibition [34, 35]. The more commission errors in our study may have partially resulted from the disturbed effect of cycling on response inhibition performance. Therefore, cycling could make the cognitive processing more automatic and reduce the performance of response inhibition while executing the color–word matching task compared with a single cognitive task.

### Neurocognitive responses on the color–word matching task

#### Early-stage neurocognitive response

P1 reflects an early-stage cognitive processing, which is usually elicited by visual stimuli in the occipitoparietal area [10]. Bullock et al*.* found a larger P1 component in performing the visual oddball task while cycling than at rest and attributed it to exercise-mediated modulation of the perceptual selection and attention process [7]. In addition, the emergence of the P1 component during the postural dual task was considered a compensatory mechanism in the elderly people, which was absent in young adults [36]. In this study, a significantly higher P1 peak amplitude in the posterior area was observed when the participants saw color–word matches while cycling than at rest. We infer that these dual tasks create compensatory visual processing and attention allocation to cope with the additional load for cycling task. In particular, P1 is an occipital neural response reflecting the operation of the extra-striate area and fusiform gyrus in the early stage [37]. This pathway in connection with the extra-striate area and fusiform gyrus also processes the early cognitive mechanisms of word, size, and color recognition [38]. Hence, showing a larger P1 is reasonable for managing the additional load added by cycling [7].

### Stimulus identification

The P3 component, first reported on the auditory oddball task [39], supported subsequent ERP studies of cognitive processing in stimulus identification [11]. P3 latency can be a measure of the speed in cognitive information processing [40]. Visual oddball tasks while cycling showed a shortening of P3 latency relative to rest [7, 13]; however, a flanker task while cycling produced a delayed P3 latency than at rest [14]. A greater demand for cognitive control on the flanker task than on the visual oddball task was suggested for this difference [7]. In this study, the relationship between P3 component and Color–Word matching task was noticed either by Xiao et al. [17]. Furthermore, in terms of the cycling effect, a delayed P3 latency was also noted during cycling than at rest when the participant was matching word’s meaning to its color. This task involves both visual and semantic processing, and thus, a delayed P3 latency was required for cognitive manipulation while cycling than at rest.

### Semantic processing

Several studies have attributed the N4 component to the demand of semantic processing [41, 42]. The N4 amplitude was modulated on the tasks involving deciding whether the word pair was semantically or phonologically related [43, 44], the color and word were semantically related [45], and numbers were arithmetically related [46]. In addition, the color–word interference continued, as evidenced by the more positive LSW [45, 47, 48]. Our study on the color–word matching task showed a smaller centrofrontal N4 and LSW while cycling compared with the resting condition. We inferred that the cycling-induced arousal may release the neural resources towards semantic processing on color–word matching.

We also found a shorter N4 peak latency on the color–word matching task while cycling than at rest. Because exercise-induced arousal was regarded as the speed up of mental cognitive processes [4], the early presence of the N4 component while cycling was also due to the similar exercise effect. Hence, the speed of internal cognitive information processing could be enhanced through cycling even with worse response’s accuracy in our color–word matching study.

### Neurocognitive responses on the spatial working memory task

Our spatial working memory study also showed a significantly higher P1 peak amplitude in the posterior area when the participants saw blue-square images during the encoding period while cycling than at rest. Similar as the inference from the color–word task, dual tasks may create compensatory visual processing and attention allocation to cope with the additional load for cycling task.

Moreover, a sustained posterior negativity (PN) when the blue squares disappeared and the participants therefore had to temporally suspend the spatial information during the retention period. Particularly, PN was significantly augmented during the late-retention stage in the resting state, indicating an increasing load on working memory with time. In the literature, the retention of spatial information was mostly characterized by PN. For examples, PN amplitude increased with spatial memory load [21] and was higher in a longer retention interval on spatial relation processing tasks [32]. The augmented PN during the late-retention period in the resting state in our study may have similar mechanism as the increased PN in accompany with the increasing working memory load or the increasing load to retain the spatial information as time. Particularly, PN augmentation was only significant in the resting state but not in the cycling state, that is, the memory load to suspend the spatial information was reduced during dual-task cycling. The reduced PN during cycling may be associated with efficient memory storage by exercise-induced compensatory memory allocation to cope with the additional load [4, 36].

### Neurocognitive responses on the arithmetic calculation task

In the present study, P1 augmentation did not occur when the participants viewed mathematical problems in which they were given a longer period (4 s) to comprehend and perform computations before answer verification compared with the pressing decision on the color–word matching task (1.5 s) and the short encoding of the uneven spatial blue squares (2 s).

Moreover, dual-task cycling yielded a smaller P3 amplitude, a more negative N4 with a shorter peak latency, and less LSW positivity while performing arithmetic computation compared with the single arithmetic computation task. We infer that simultaneous cycling and arithmetic computing generated a sequence of cognitive mechanisms including a less cognitive demand for identifying the problem, more semantic processing with a faster response to commence mental arithmetic calculations, and consequential decrement of cerebral activation in subsequent calculations. As for the late neurocognitive recruitment, positive LSW, particularly in the parietal area has been demonstrated to be a significant indicator for arithmetic calculations, modulated by the problem size and arithmetic strategy [19, 20], and decreased with practice [49]. These findings support our inference that less positive LSW while cycling than at rest was associated with the decrement of cerebral activation while performing arithmetic computation and cycling simultaneously.

### Different ERP responses among various neurocognitive tasks

Cycling elicited different ERP responses across the three tasks. A more pronounced P1 response was observed on both the spatial memory and color–word matching tasks, but not on the arithmetic calculation task. Additionally, a delayed P3 response and a smaller P3 amplitude were noted on the color–word matching task and the arithmetic calculation task, respectively. Furthermore, the color–word matching task displayed reduced N4, while the arithmetic calculation task exhibited an enhanced N4 response.

Indeed, the visual demands of the spatial memory and color–word matching tasks align with the observed P1 findings. Furthermore, the delayed or smaller P3 response respectively on the color–word matching and arithmetic calculation tasks indicates less efficient cognitive load management. Additionally, the varied N4 responses reflect the semantic demands of the color–word matching and arithmetic calculation tasks. Hence, taking into account the impact of cycling relies on the distinctive characteristics of various tasks.

### Comparing ERP responses with previous studies

Previous research has delved into ERP responses within tasks such as visual oddball and flanker paradigms, conducted during cycling in contrast to a state of rest. These tasks primarily delve into fundamental facets of attention and perceptual capacity, which diverge somewhat from the conceptual and semantic levels that our current tasks aim to address. However, it is worth noting that the visual oddball tasks undertaken by Bullock et al*.* [7] and Yagi et al*.* [13] could potentially lend support to the ERP responses evoked by cycling (evident in heightened P1 and diminished P3) as discovered in our investigations.

In the study by Bullock et al*.* [7], cycling induced a heightened P1 response during the visual oddball task, similar to the augmented P1 seen in the color–word matching and spatial working memory tasks. Additionally, it is noteworthy that Bullock et al*.* also utilized a low-intensity cycling protocol, similar to ours. Furthermore, their visual oddball task, much like our color–word matching and spatial working memory tasks, necessitates a heightened visual demand. Hence, we posit that cycling potentially exerts a modulatory influence on neural information processing during the initial stages of sensory processing, a notion also acknowledged by Bullock et al.

In the research conducted by Yagi et al*.* [13], cycling led to a reduction in the P3 response during the auditory and visual oddball tasks, similar to the attenuated P3 observed in the arithmetic calculation task. Interestingly, this decrease in attentional allocation (reflected by the smaller P3) was accompanied by an accelerated cognitive information processing speed (manifesting as an earlier P3), faster reaction times, and a higher error rate while pedaling. These findings prompted Yagi et al*.* to suggest that the impact of moderate aerobic pedaling on cognitive performance does not equate to a global cognitive enhancement. In our study, although we did not identify disparities in error rates or reaction times between the cycling and resting states, we observed a reduction in the P3 response. Consequently, our conjecture is that there might have been a depletion in the allocation of attentional resources while dual-task cycling.

### Benefit of cycling on the dual cognitive-motor neurocognitive investigations and possible neural mechanisms

Cycling offers distinct advantages over walking and running when studying EEG and ERPs. One key benefit is the ability of participants to maintain a consistent posture throughout the activity, eliminating the need for frequent changes in location. Additionally, while cycling, participants have a clear and stable view of the neurocognitive task images displayed on a securely positioned computer monitor in front of them. This setup facilitates the execution of diverse experiments with enhanced stability and precision.

In this study, we implemented a modified cycling protocol involving light-intensity cycling, with a specific emphasis on maintaining a consistent power output of 30 W and a pedaling speed of 40 rpm. The cycling durations for the color–word, arithmetic calculation, and spatial memory tasks were set at 7, 16, and 20 min, respectively. Following this specific protocol, the study's outcomes were subsequently identified and analyzed.

To start and maintain exercises, various peripheral and central physiological systems are engaged, consequently leading to the augmentation of motor and associated brain regions. Nonetheless, the brain must modulate activity in regions that are not directly tied to exercise in order to facilitate the aforementioned exercise-related systems, resulting in reduced responsiveness within these regions (Olson et al*.*, 2016; Yagi et al*.*, 1999) [8, 13]. Moreover, specific non-exercise areas could display increased activation in a compensatory manner to support cognitive performance, which could potentially be affected by exercising. This phenomenon is illustrated by the observed P1 enhancement in the posterior region during color–word matching and spatial working memory tasks in our study. Similar P1 findings were also reported in a study by Bullock et al*.* [7] involving a visual oddball task.

### Limitation of study

In this study, the road was moving at a constant speed while performing a single cognitive task. The reason for this display is to shorten the difference in visual environment with dual-task cycling where the moving of the road was controlled by participant’s pedaling. Performing cognitive test while perceiving that the road is moving is not a perfect single task. It creates a limitation when trying to explain solely cognitive effect. Nevertheless, significant differences between cycling and rest were still demonstrated in the ERP components used to explain the dual-task effect.

### Future directions for dual-task effects on neurocognitive functioning

The ERP difference between the dual cognitive–walking task and single cognitive task demonstrated the impact of physiological recruitment by aging and disease [22–24]. A study on LED-flashing detection while walking demonstrated a prolonged P1 latency and a reduced P1 amplitude compared with the same task being executed while standing; this prolonging was significant in younger participants, but the amplitude reduction was significant in older participants [24]. In another study, individuals with Parkinson’s disease exhibited a significant reduction in the P3 amplitude from standing to walking with the auditory oddball task, but the reduction was nonsignificant in the healthy controls [22]. Different demands of cognitive workload and postural control between cycling and walking led to various dual-task cycling effects. In this study, several dual-task effects were demonstrated by the enhanced visual processing and reduced semantic processing on the color–word matching task, the reduced cognitive and semantic processing during the arithmetic computation, and the enhanced visual processing and the release of memory load during the spatial working memory task. The proposed dual-task cycling is also useful for developing age-related and disease-specific neurocognitive markers and deserves in-depth investigation.

## Conclusion

The behavior analysis revealed that cycling resulted in a shorter reaction time with an approaching significance level on the spatial working memory task, whereas ERPs showed different aspects of cognitive processing on various neurocognitive tasks. First, simultaneous cognitive processing while cycling may create an additional load for cycling task, and therefore, more visual processing is required for the color–word image or blue-square image (evidenced by a greater P1 amplitude and a delayed P3 latency) to identify the color–word. Second, dual-task cycling could reduce the demand of poststimulus semantic processing on color–word matching (evidenced by a shorter N4 latency, a less negative N4, and a less positive LSW) and the memory load to temporally suspend the spatial information on the spatial memory task (evidenced by a less positive LSW). Third, simultaneous arithmetic computing and cycling required less cognitive demand for identifying the problem, more semantic processing (a more negative N4) to commence mental arithmetic calculation, and compensatory decrement of cerebral activation (less positive LSW) in the subsequent calculation. Based on the findings of the present study, specific aspects of cognitive processing demonstrated cognitive improvement, while others showed the requirement of the allocation of supplementary resources. Consequently, cycling appears to enhance particular dimensions of cognitive processes, such as semantic processing and memory retention, while leaving other aspects unaffected or potentially disrupted.

## Data Availability

The datasets generated during and/or analyzed during the current study are available from the corresponding author on reasonable request.

## References

[1] Hötting K, Röder B (2013). Beneficial effects of physical exercise on neuroplasticity and cognition. Neurosci Biobehav Rev.

[2] Bamidis PD, Vivas AB, Styliadis C, Frantzidis C, Klados M, Schlee W, Siountas A, Papageorgiou SG (2014). A review of physical and cognitive interventions in aging. Neurosci Biobehav Rev.

[3] Anderson-Hanley C, Arciero PJ, Brickman AM, Nimon JP, Okuma N, Westen SC, Merz ME, Pence BD, Woods JA, Kramer AF (2012). Exergaming and older adult cognition: A cluster randomized clinical trial. Am J Prev Med.

[4] Lambourne K, Tomporowski P (2010). The effect of exercise-induced arousal on cognitive task performance: A meta-regression analysis. Brain Res.

[5] Hazamy AA, Altmann LJP, Stegemöller E, Bowers D, Lee HK, Wilson J, Okun MS, Hass CJ (2017). Improved cognition while cycling in Parkinson’s disease patients and healthy adults. Brain Cogn.

[6] Chang H-C, Chen C-C, Liaw J-W, Chiou W-D, Weng Y-H, Chang Y-J, Lu C-S (2020). The effects of dual-task in patients with Parkinson's disease performing cognitive-motor paradigms. J Clin Neurosci.

[7] Bullock T, Cecotti H, Giesbrecht B (2015). Multiple stages of information processing are modulated during acute bouts of exercise. Neuroscience.

[8] Olson RL, Chang Y-K, Brush CJ, Kwok AN, Gordon VX, Alderman BL (2016). Neurophysiological and behavioral correlates of cognitive control during low and moderate intensity exercise. Neuroimage.

[9] Lin C-T, King J-T, John AR, Huang K-C, Cao Z, Wang Y-K: The impact of vigorous cycling exercise on visual attention: A study with the BR8 wireless dry EEG system. 2021, 15. 10.3389/fnins.2021.621365 PMC7928413 33679304

[10] Jeffreys DA, Axford JG (1972). Source locations of pattern-specific components of human visual evoked potentials. II. Component of extrastriate cortical origin. Exp Brain Res.

[11] Patel SH, Azzam PN (2005). Characterization of N200 and P300: Selected studies of the event-related potential. Int J Med Sci.

[12] Scanlon JEM, Sieben AJ, Holyk KR, Mathewson KE (2017). Your brain on bikes: P3, MMN/N2b, and baseline noise while pedaling a stationary bike. Psychophysiology.

[13] Yagi Y, Coburn KL, Estes KM, Arruda JE (1999). Effects of aerobic exercise and gender on visual and auditory P300, reaction time, and accuracy. Eur J Appl Physiol Occup Physiol.

[14] Pontifex MB, Hillman CH (2007). Neuroelectric and behavioral indices of interference control during acute cycling. Clin Neurophysiol.

[15] Montemayor C, Haladjian HH (2017). Perception and cognition are largely independent, but still affect each other in systematic ways: Arguments from evolution and the consciousness-attention dissociation. Front Psychol.

[16] Atkinson CM, Drysdale KA, Fulham WR (2003). Event-related potentials to Stroop and reverse Stroop stimuli. Int J Psychophysiol.

[17] Xiao X, Qiu J, Zhang Q: The dissociation of neural circuits in a Stroop task. NeuroReport 2009, 20(7). 10.1097/WNR.0b013e32832a0a10 19349920

[18] Lurquin JH, McFadden SL, Harbke CR (2014). An electrophysiological investigation of the effects of social rejection on self control. J Soc Psychol.

[19] Núñez-Peña MI, Cortiñas M, Escera C: Problem size effect and processing strategies in mental arithmetic. Neuroreport 2006, 17(4). 10.1097/01.wnr.0000203622.24953.c2 16514358

[20] Núñez-Peña MI, Gracia-Bafalluy M, Tubau E (2011). Individual differences in arithmetic skill reflected in event-related brain potentials. Int J Psychophysiol.

[21] Mecklinger A, Pfeifer E (1996). Event-related potentials reveal topographical and temporal distinct neuronal activation patterns for spatial and object working memory. Cogn Brain Res.

[22] Maidan I, Fahoum F, Shustak S, Gazit E, Patashov D, Tchertov D, Giladi N, Hausdorff JM, Mirelman A (2019). Changes in event-related potentials during dual task walking in aging and Parkinson's disease. Clin Neurophysiol.

[23] De Sanctis P, Malcolm BR, Mabie PC, Francisco AA, Mowrey WB, Joshi S, Molholm S, Foxe JJ (2020). Mobile Brain/Body Imaging of cognitive-motor impairment in multiple sclerosis: Deriving EEG-based neuro-markers during a dual-task walking study. Clin Neurophysiol.

[24] Protzak J, Wiczorek R, Gramann K (2021). Peripheral visual perception during natural overground dual-task walking in older and younger adults. Neurobiol Aging.

[25] Faul F, Erdfelder E, Lang AG, Buchner A (2007). G*Power 3: a flexible statistical power analysis program for the social, behavioral, and biomedical sciences. Behav Res Methods.

[26] Jensen PS, Kenny DT (2004). The effects of yoga on the attention and behavior of boys with Attention-Deficit/hyperactivity Disorder (ADHD). J Atten Disord.

[27] Zeng N, Pope Z, Gao Z (2017). Acute effect of virtual reality exercise bike games on college students' physiological and psychological outcomes. Cyberpsychol Behav Soc Netw.

[28] Delorme A, Makeig S (2004). EEGLAB: an open source toolbox for analysis of single-trial EEG dynamics including independent component analysis. J Neurosci Methods.

[29] Melinder A, Gredebäck G, Westerlund A, Nelson CA (2010). Brain activation during upright and inverted encoding of own- and other-age faces: ERP evidence for an own-age bias. Dev Sci.

[30] Winograd MR, Rosenfeld JP (2011). Mock crime application of the Complex Trial Protocol (CTP) P300-based concealed information test. Psychophysiology.

[31] Tan M, Wyble B (2015). Understanding how visual attention locks on to a location: Toward a computational model of the N2pc component. Psychophysiology.

[32] van der Ham IJ, van Strien JW, Oleksiak A, van Wezel RJ, Postma A (2010). Temporal characteristics of working memory for spatial relations: an ERP study. Int J Psychophysiol.

[33] Herold F, Hamacher D, Törpel A, Goldschmidt L, Müller NG, Schega L (2020). Does squatting need attention?-A dual-task study on cognitive resources in resistance exercise. PLoS ONE.

[34] Helenius P, Laasonen M, Hokkanen L, Paetau R, Niemivirta M (2010). Neural correlates of late positivities associated with infrequent visual events and response errors. Neuroimage.

[35] Sucec J, Herzog M, Van Diest I, Van den Bergh O, von Leupoldt A (2018). The impairing effect of dyspnea on response inhibition. Int J Psychophysiol.

[36] Yu S-H, Hwang I-S, Huang C-Y (2018). Neuronal responses to a postural dual-task with differential attentional prioritizations: Compensatory resource allocation with healthy aging. J Gerontol B.

[37] Di Russo F, Aprile T, Spitoni G, Spinelli D (2008). Impaired visual processing of contralesional stimuli in neglect patients: a visual-evoked potential study. Brain.

[38] Allison T, McCarthy G, Nobre A, Puce A, Belger A (1994). Human extrastriate visual cortex and the perception of faces, words, numbers, and colors. Cereb Cortex.

[39] Sutton S, Braren M, Zubin J, John ER (1965). Evoked-potential correlates of stimulus uncertainty. Science.

[40] Kutas M, McCarthy G, Donchin E (1977). Augmenting mental chronometry: the P300 as a measure of stimulus evaluation time. Science.

[41] Kutas M, Federmeier KD (2000). Electrophysiology reveals semantic memory use in language comprehension. Trends Cogn Sci.

[42] Kutas M, Federmeier KD (2010). Thirty years and counting: Finding meaning in the N400 component of the event-related brain potential (ERP). Annu Rev Psychol.

[43] Khateb A, Pegna AJ, Landis T, Mouthon MS, Annoni J-M (2010). On the origin of the N400 effects: An ERP waveform and source localization analysis in three matching tasks. Brain Topogr.

[44] Nogueira AML, Bueno OFA, Manzano GM, Kohn AF, Pompéia S: Late positive slow waves as markers of chunking during encoding. Front Psychol 2015, 6. 10.3389/fpsyg.2015.01032 PMC4516824 26283984

[45] Ergen M, Saban S, Kirmizi-Alsan E, Uslu A, Keskin-Ergen Y, Demiralp T (2014). Time–frequency analysis of the event-related potentials associated with the Stroop test. Int J Psychophysiol.

[46] Galfano G, Mazza V, Angrilli A, Umiltà C (2004). Electrophysiological correlates of stimulus-driven multiplication facts retrieval. Neuropsychologia.

[47] Liotti M, Woldorff MG, Perez R, Mayberg HS (2000). An ERP study of the temporal course of the Stroop color-word interference effect. Neuropsychologia.

[48] Hanslmayr S, Pastötter B, Bäuml K-H, Gruber S, Wimber M, Klimesch W (2007). The electrophysiological dynamics of interference during the Stroop task. J Cogn Neurosci.

[49] Núñez-Peña MI (2008). Effects of training on the arithmetic problem-size effect: an event-related potential study. Exp Brain Res.

